# Supplementary material for: Black carbon-climate interactions regulate dust burdens over India revealed during COVID-19
Source: Nat Commun. 2022 Apr 5;13:1839. doi: 10.1038/s41467-022-29468-1 (PMC8983761; doi:10.1038/s41467-022-29468-1)
Supplement: Supplementary file 1 — Supplementary Information [file 41467_2022_29468_MOESM1_ESM.pdf]

# Supplementary Information

## Black Carbon-Climate Interactions Regulate Dust Burdens over India Revealed during COVID-19

Linyi Wei[1†], Zheng Lu[2†], Yong Wang[1*], Xiaohong Liu[2*], Weiyi Wang[3,4], Chenglai Wu[3], Xi Zhao[2], Stefan Rahimi[5], Wenwen Xia[1], and Yiquan Jiang[6]

[1]Department of Earth System Science, Ministry of Education Key Laboratory for Earth System Modeling, Institute for Global Change Studies, Tsinghua University, Beijing 100084, China.

[2]Department of Atmospheric Sciences, Texas A&M University, College Station, TX 77843, USA.

[3]International Center for Climate and Environment Sciences, Institute of Atmospheric Physics, Chinese Academy of Sciences, Beijing, 100029, China.

[4]University of Chinese Academy of Sciences, Beijing, 100049, China.

[5]Institute of the Environment and Sustainability, University of California Los Angeles, Los Angeles, CA 90095, USA.

[6]CMA-NJU Joint Laboratory for Climate Prediction Studies, Institute for Climate and Global Change Research, School of Atmospheric Sciences, Nanjing University, Nanjing, China.

* Correspondence to: X.L. (xiaohong.liu@tamu.edu) and Y.W. (yongw@mail.tsinghua.edu.cn)

† These authors contributed equally to this work.

**Supplementary Information includes:**

Supplementary Fig. 1-Fig. 20

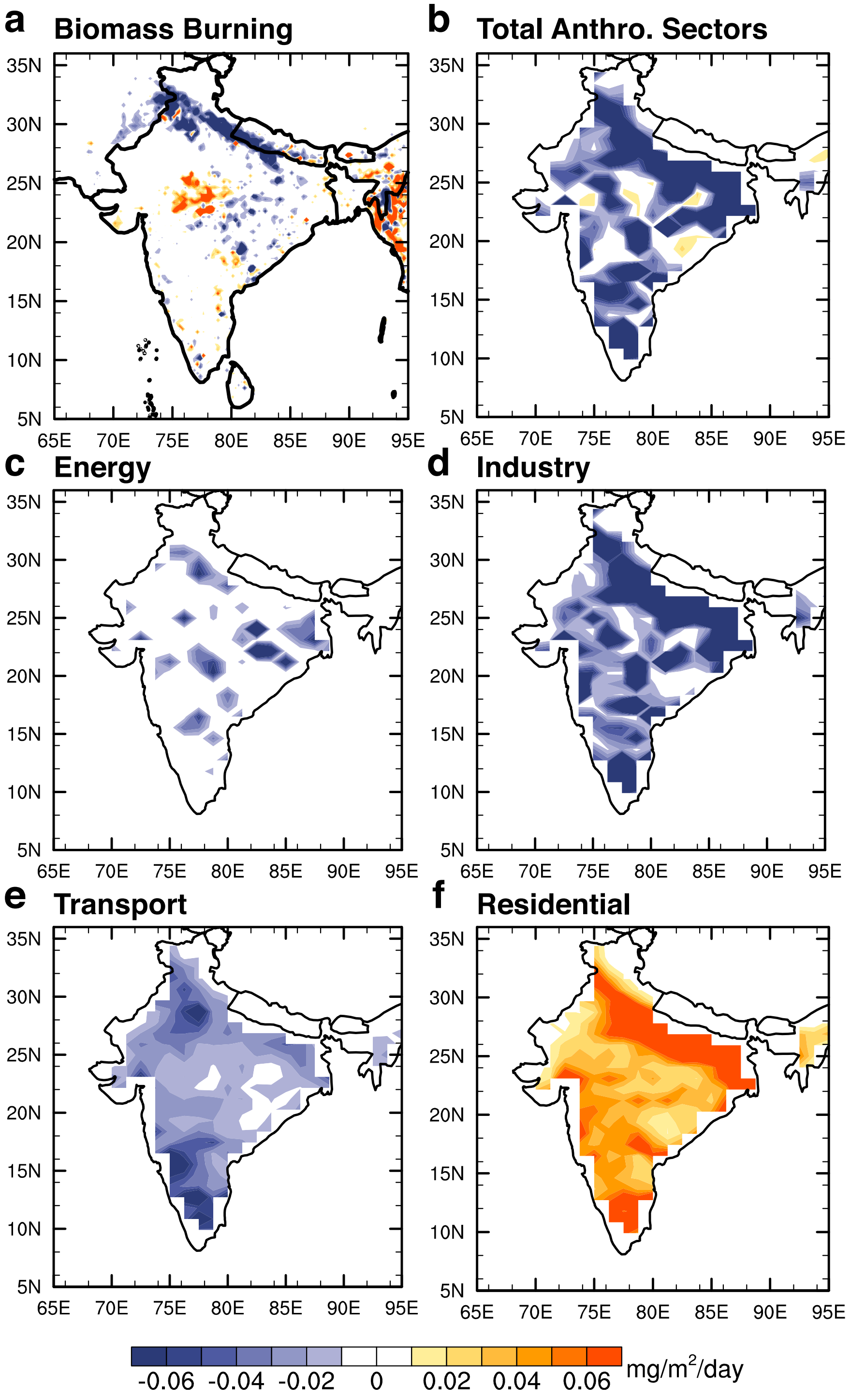


**Supplementary Figure 1. BC emission changes.** (**a**) BC emission change in April-May between 2020 and 2015-2019 for biomass burning from GFED4. (**b-f**) BC emission changes in April-May 2020 due to lockdown for anthropogenic sectors (not including biomass burning) of (**b**) total, (**c**) energy, (**d**) industry, (**e**) transport, and (**f**) residential from Supplementary ref [1,2].

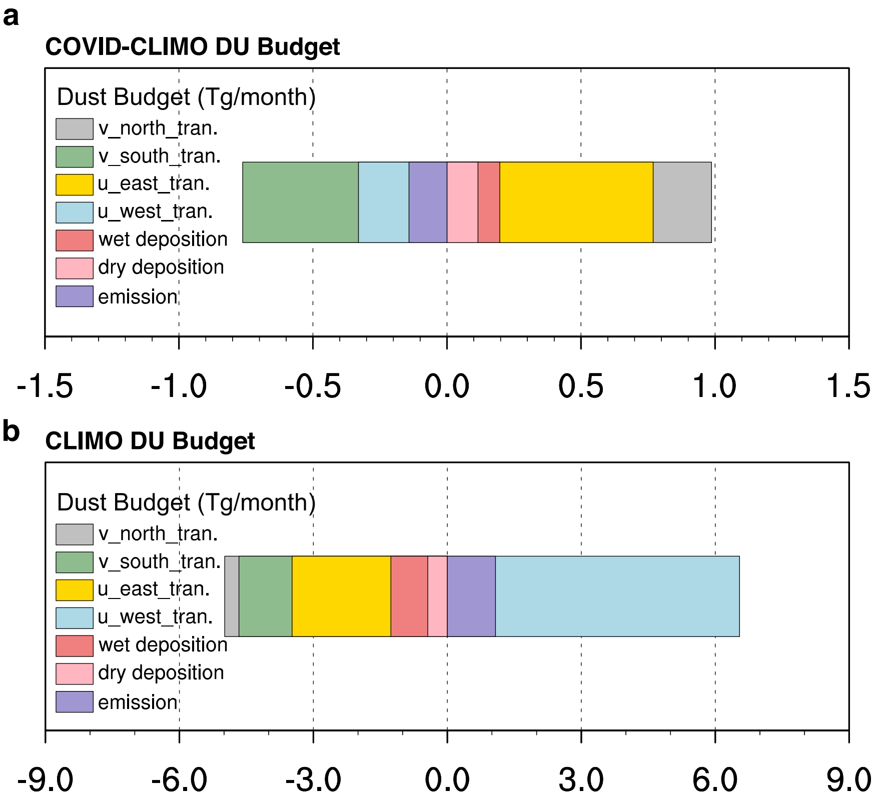


**Supplementary Figure 2. Northern Indian dust budget in MERRA-2.** Local emissions, transport across the west (70˚E), east (88˚E), south (25˚N), north (35˚N) boundaries, dry deposition and wet deposition respectively for (**a**) differences between 2020 (COVID) and 2015-2019 (CLIMO) and (**b**) climatological means of 2015-2019. For transport, transport into the region is positive otherwise negative. Note the different scales between (a) and (b).

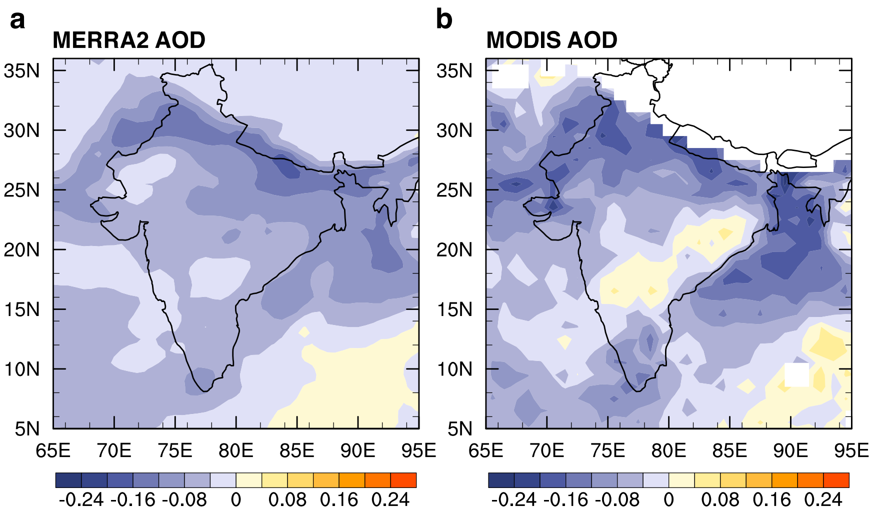


**Supplementary Figure 3. AOD changes.** AOD (550 nm) differences in April-May between 2020 and 2015-2019 for (**a**) MERRA-2 and (**b**) MODIS.

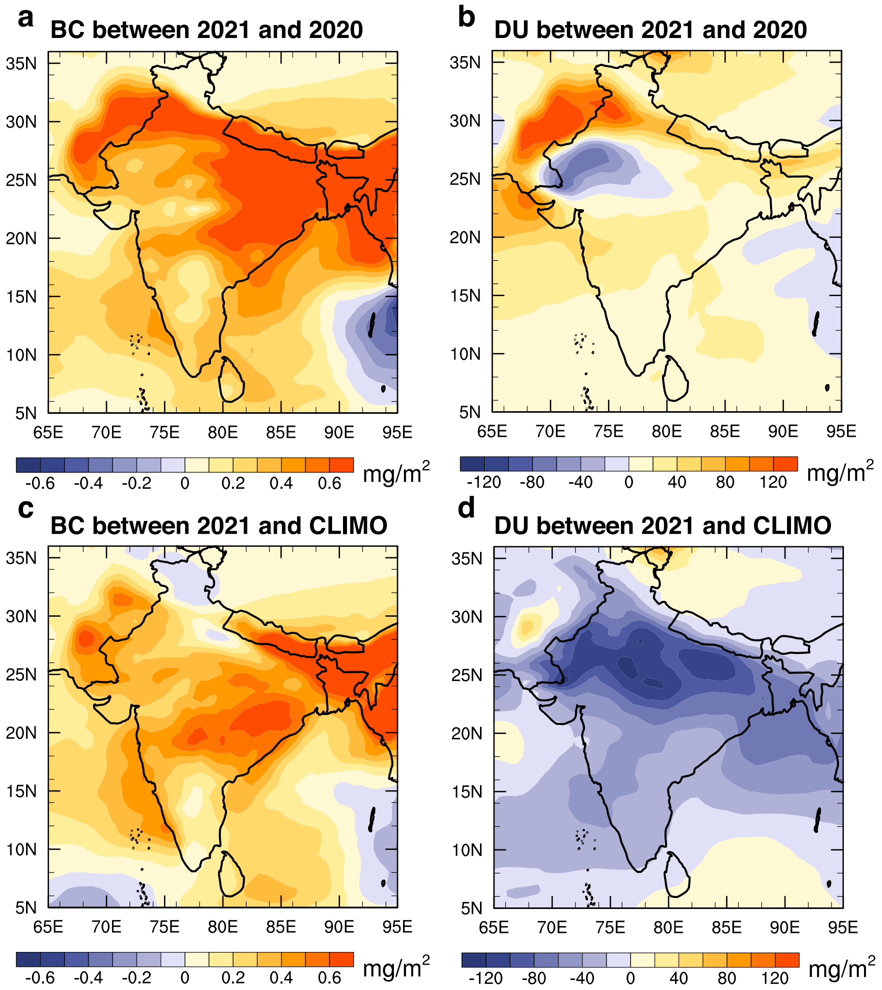


**Supplementary Figure 4. BC and DU burden changes in 2021.** Differences of MERRA-2 (**a, c**) BC and (**b, d**) DU burdens in April-May (**a, b**) between 2021 and 2020 and (**c, d**) between 2021 and 2015-2019.

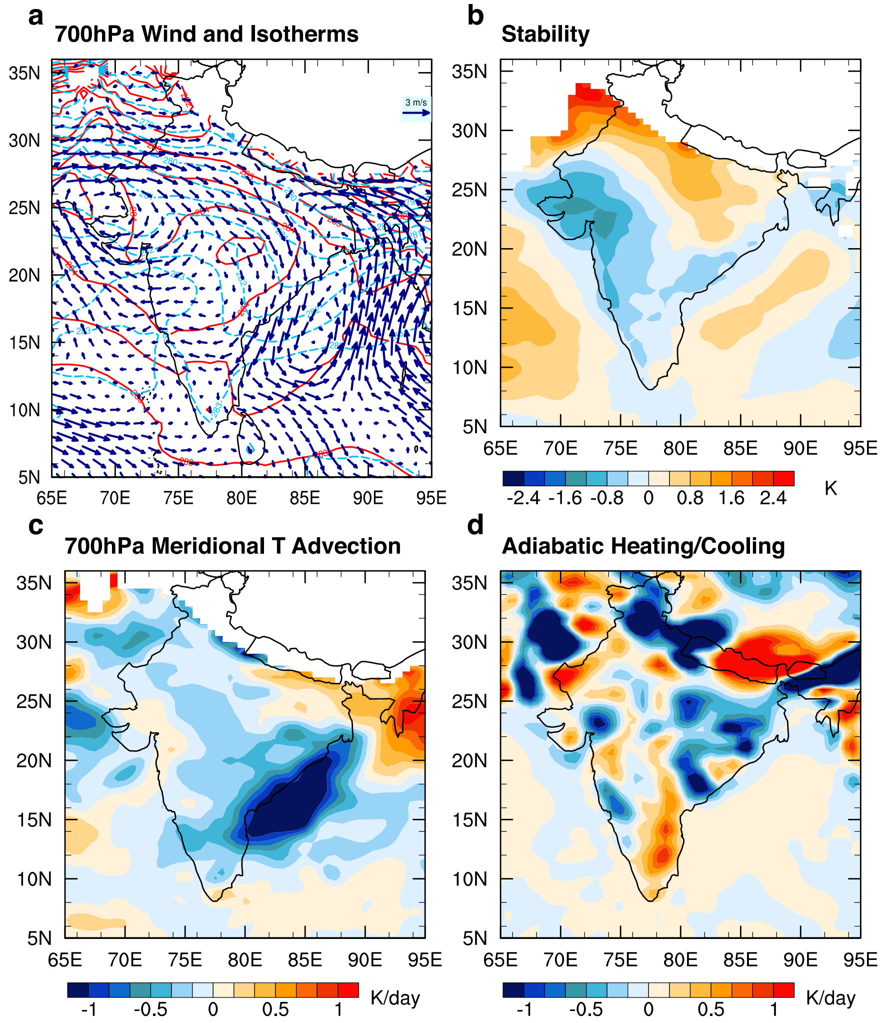


**Supplementary Figure 5. Changes of winds at 700 hPa, atmospheric stability, meridional temperature advection and adiabatic heating/cooling near the surface pressure in April-May.** (**a**) MERRA-2 700 hPa winds superimposed by air temperatures at 700 hPa in 2020 (blue) and 2015-2019 (red), (**b**) atmospheric stability, (**c**) 700 hPa meridional temperature advection and (**d**) adiabatic heating/cooling at the lowest pressure level above the surface.

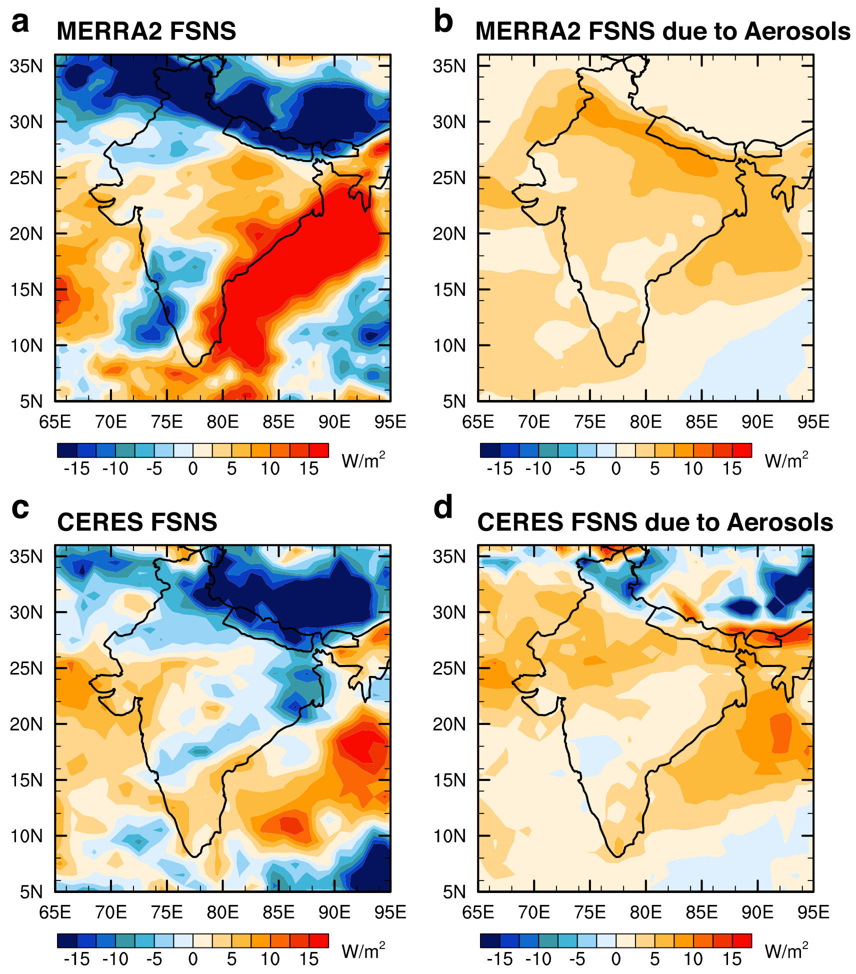


**Supplementary Figure 6. Net shortwave radiative effect at the surface (FSNS) changes.** Differences of FSNS (**a, c**) due to both aerosols and clouds, and (**b, d**) due to aerosols in April-May between 2020 and 2015-2019 for (**a, b**) MERRA-2 and (**c, d**) CERES.

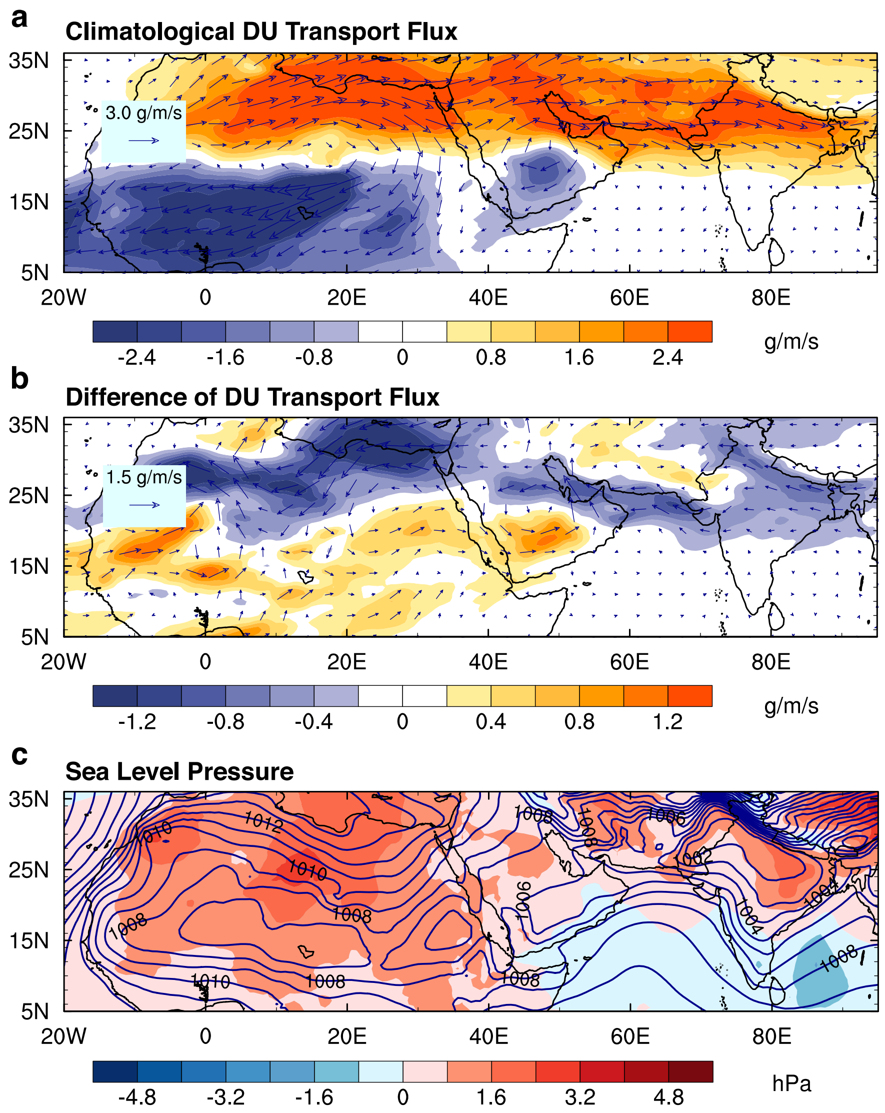


**Supplementary Figure 7. (a) 2015-2019 mean of DU transport flux in April-May. (b-c) Changes of (b) DU transport flux by zonal wind and (c) sea level pressure (SLP) in April-May between 2020 and 2015-2019 superposed by 2015-2019 April-May mean of SLP.**

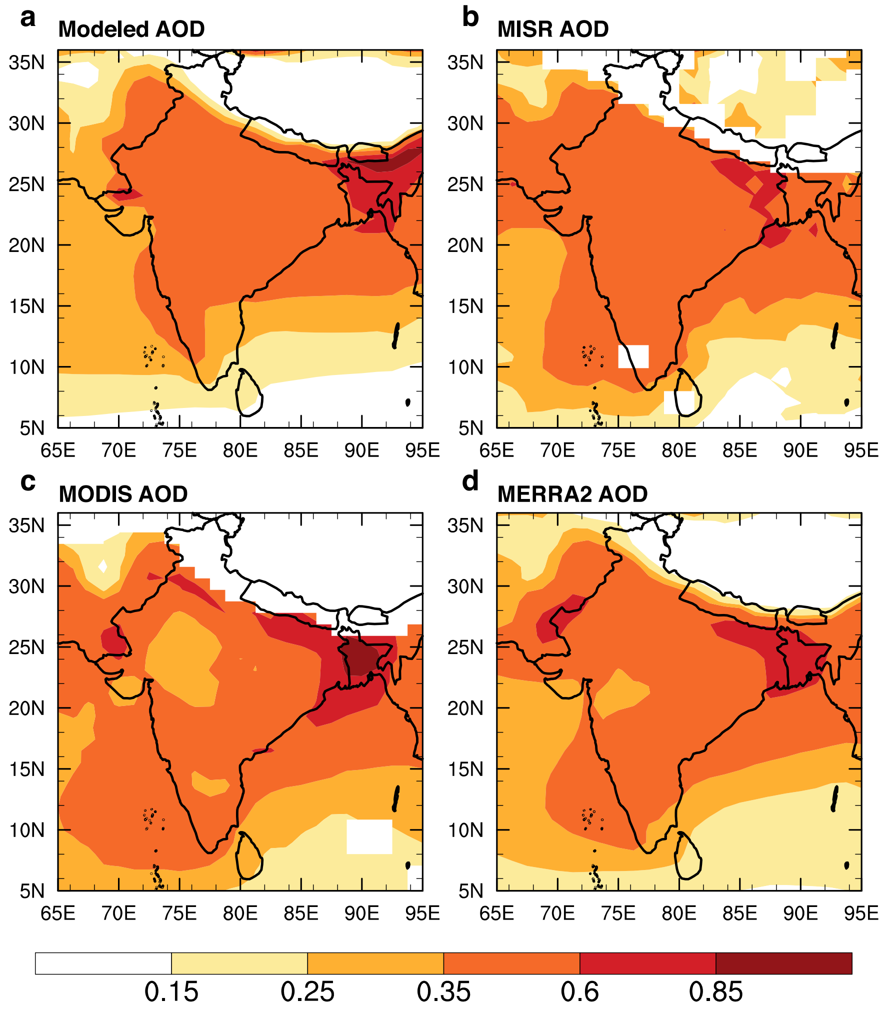


**Supplementary Figure 8. Climatological AOD.** (**a**) CESM2, (**b**) MISR, (**c**) MODIS and (**d**) MERRA-2.

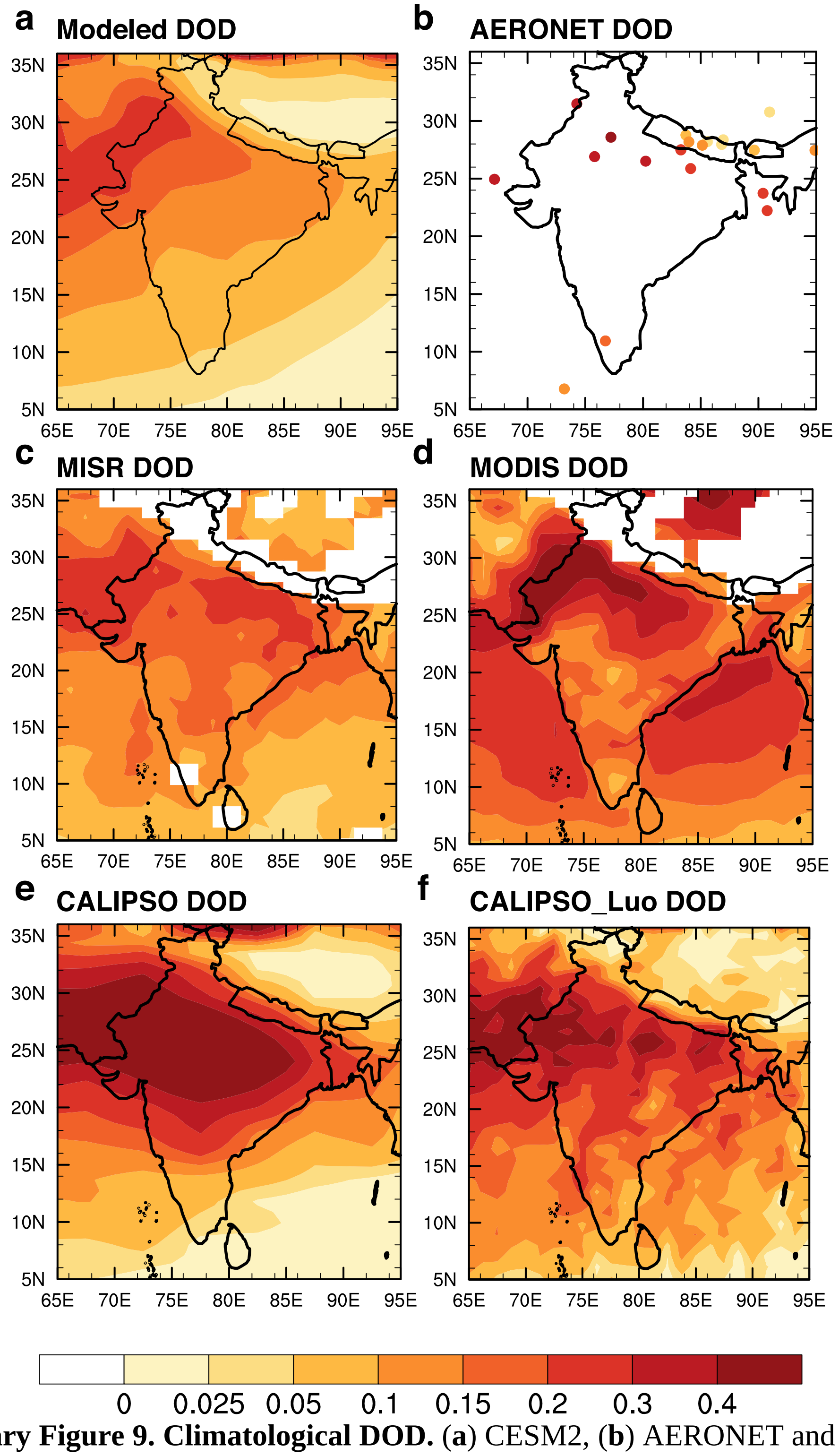


**Supplementary Figure 9. Climatological DOD.** (**a**) CESM2, (**b**) AERONET and (**c**) MISR (for coarse mode aerosols), (**d**) MODIS[3], (**e**) CALIPSO and (**f**) CALIPSO by Luo[4,5].

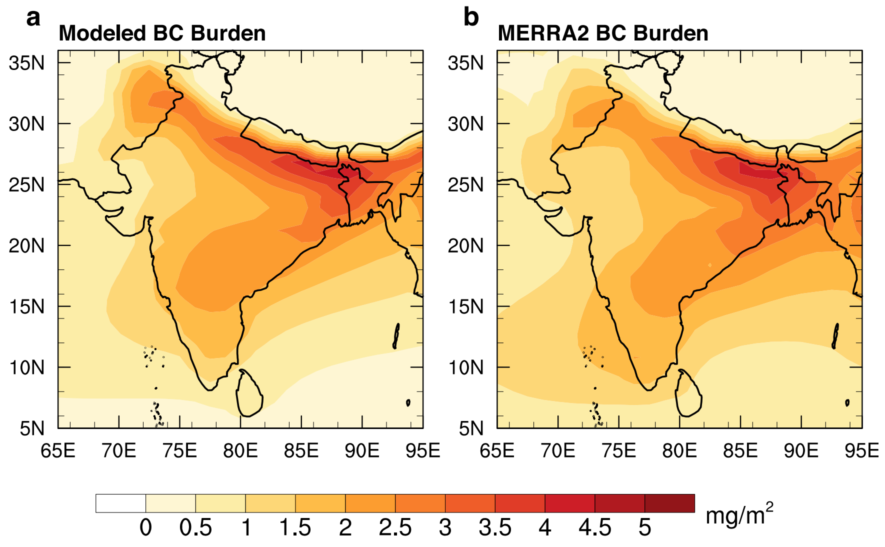


**Supplementary Figure 10. Climatological BC burdens. (a)** CESM2 and **(b)** MERRA-2.

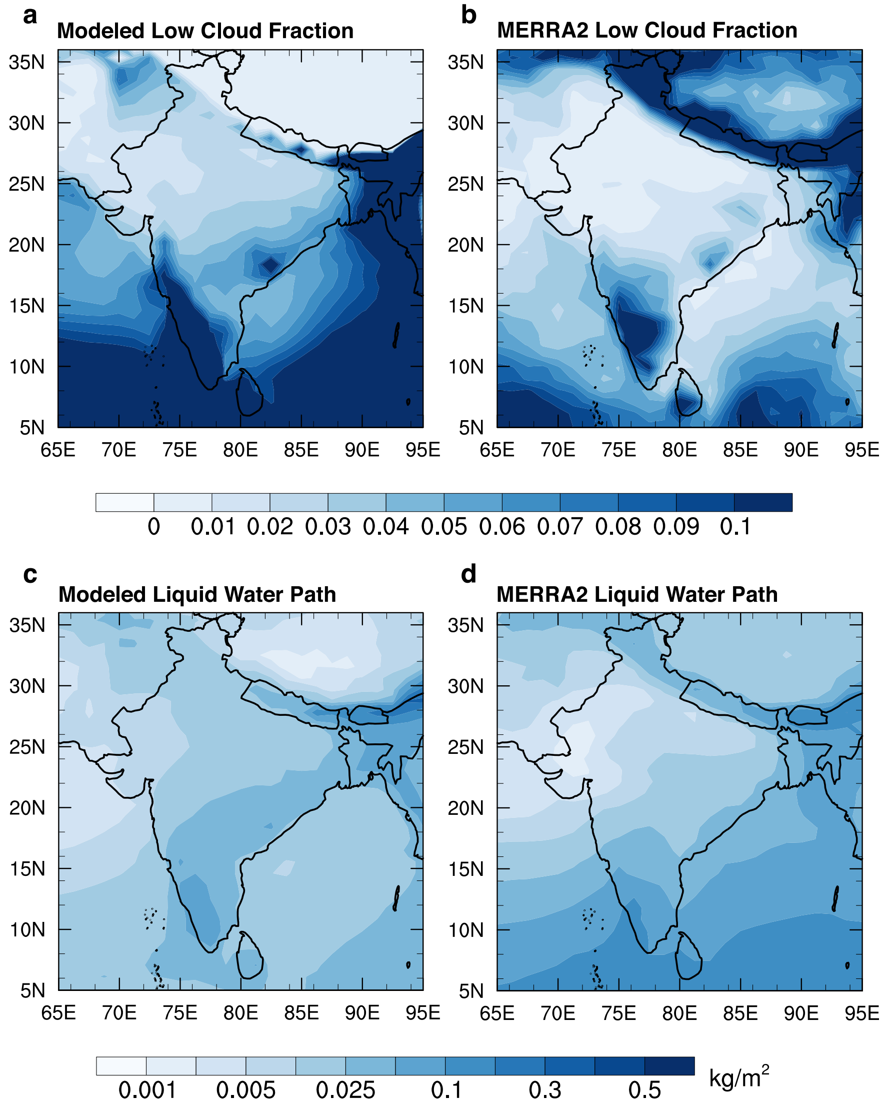


**Supplementary Figure 11. Climatological low cloud fraction and liquid water path.** (**a, b**) Low cloud fraction and (**c, d**) liquid water path for (**a, c**) CESM2 and (**b, d**) MERRA-2.

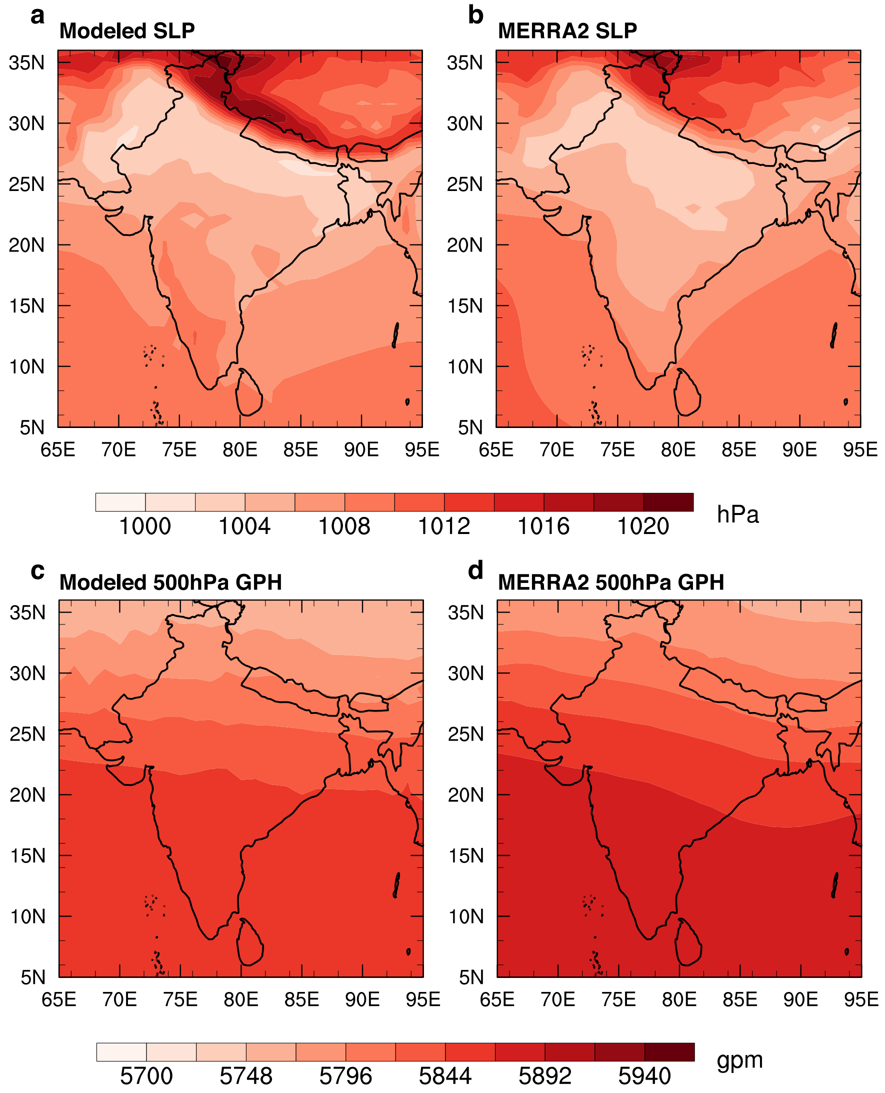


**Supplementary Figure 12. Climatological (a, b) sea level pressure (SLP) and (c, d) 500 hPa geopotential height (GPH) for (a, c) CESM2 and (b, d) MERRA2.**

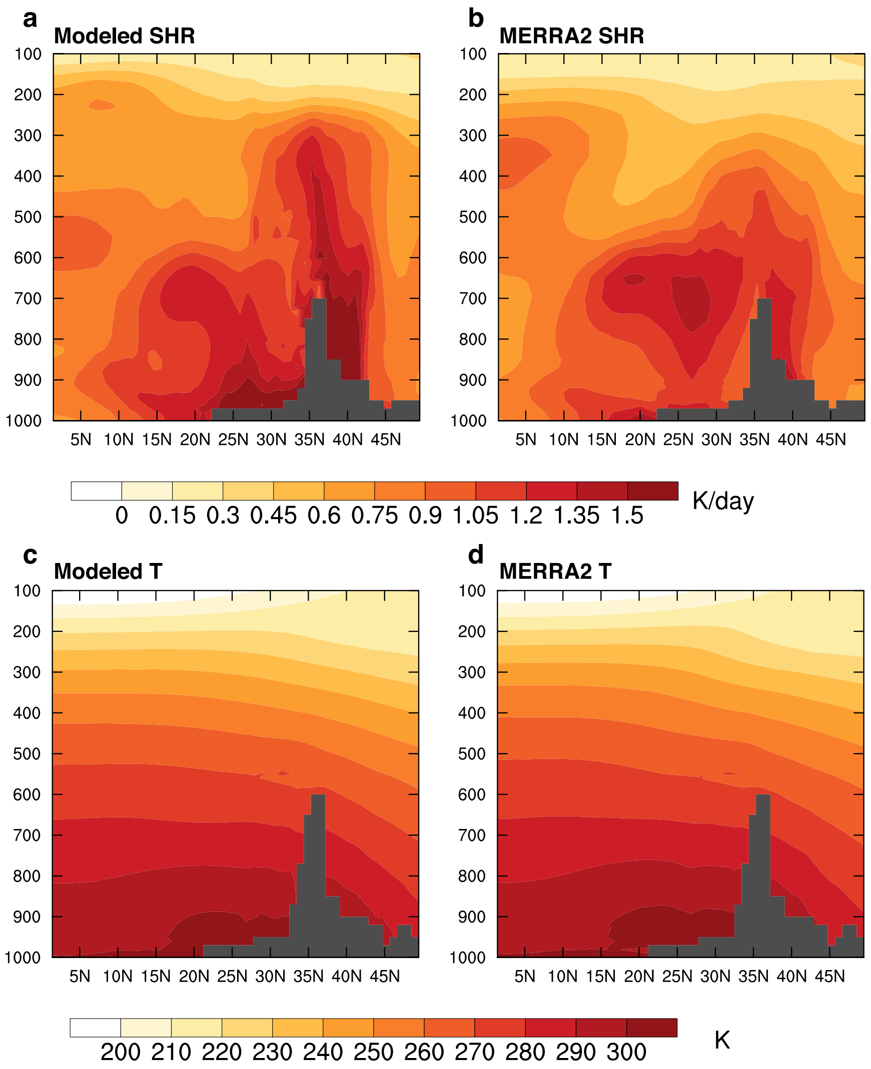


**Supplementary Figure 13. Climatological zonal mean (a, b) solar heating rate (SHR) and (c, d) air temperature (T) over (70°E–90°E) for (a, c) CESM2 and (b, d) MERRA2.**

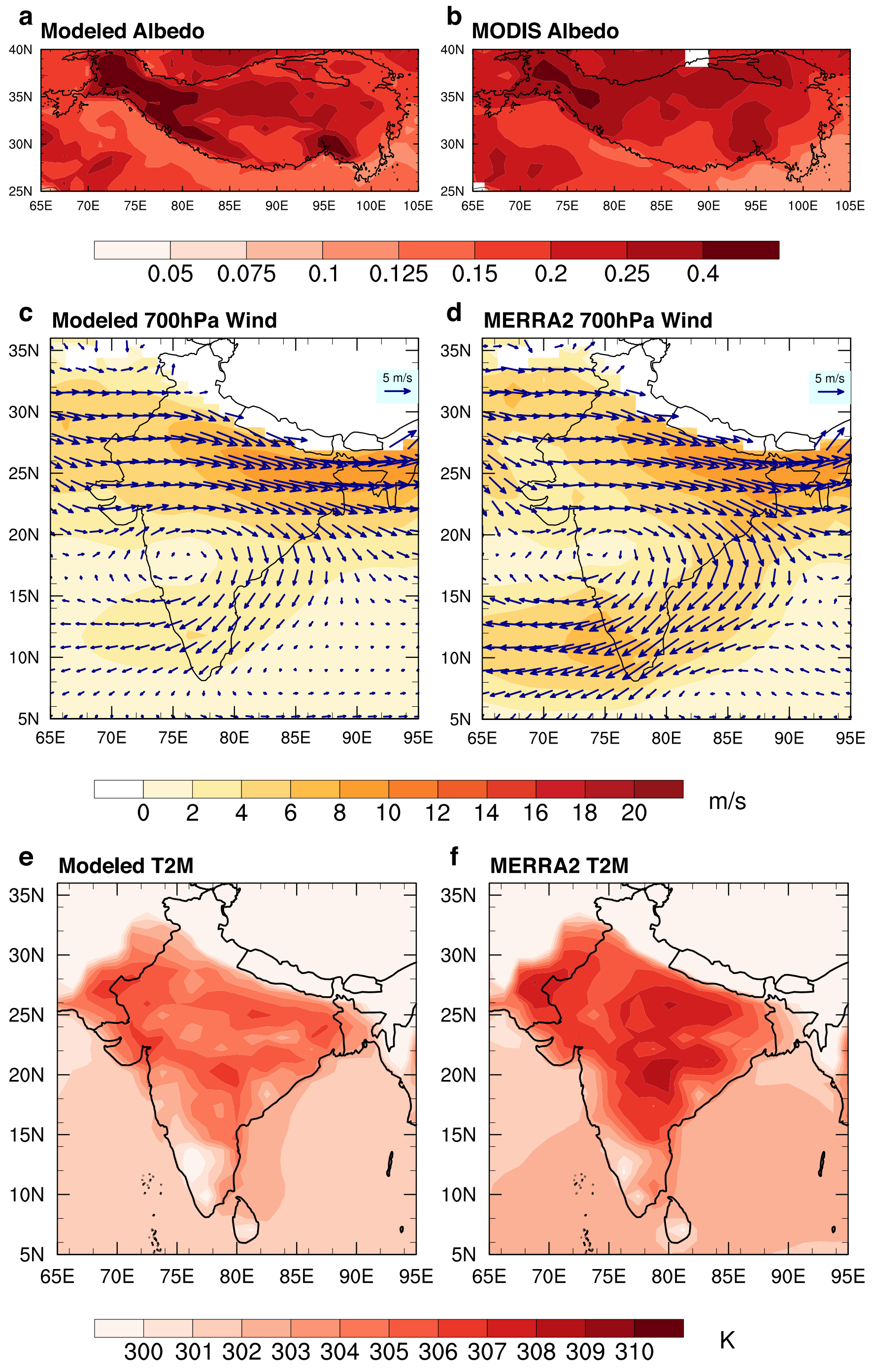


**Supplementary Figure 14. Climatological (a, b) surface albedo, (c, d) 700hPa winds and (e, f) 2m air temperature for (a, c, e) CESM2, (b) MODIS and (d, f) MERRA2.**

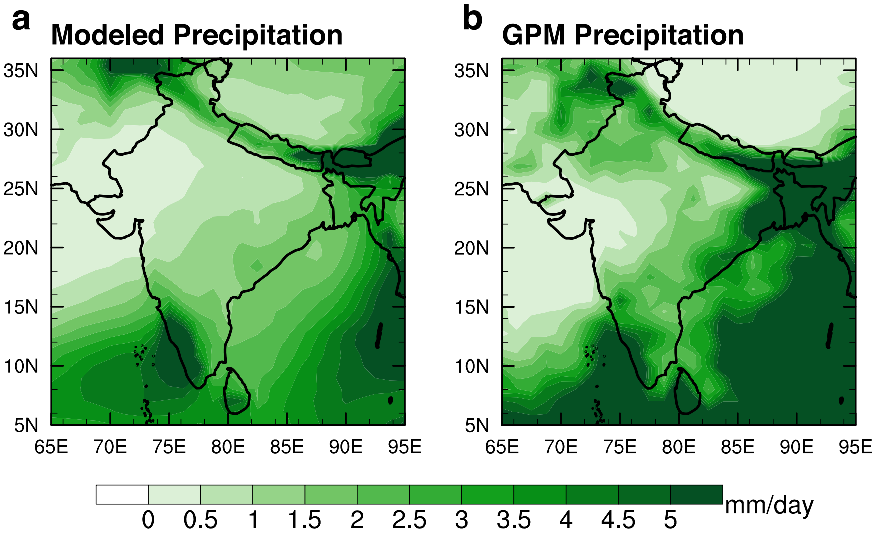


**Supplementary Figure 15. Climatological (a) modeled and (b) observed precipitation.**

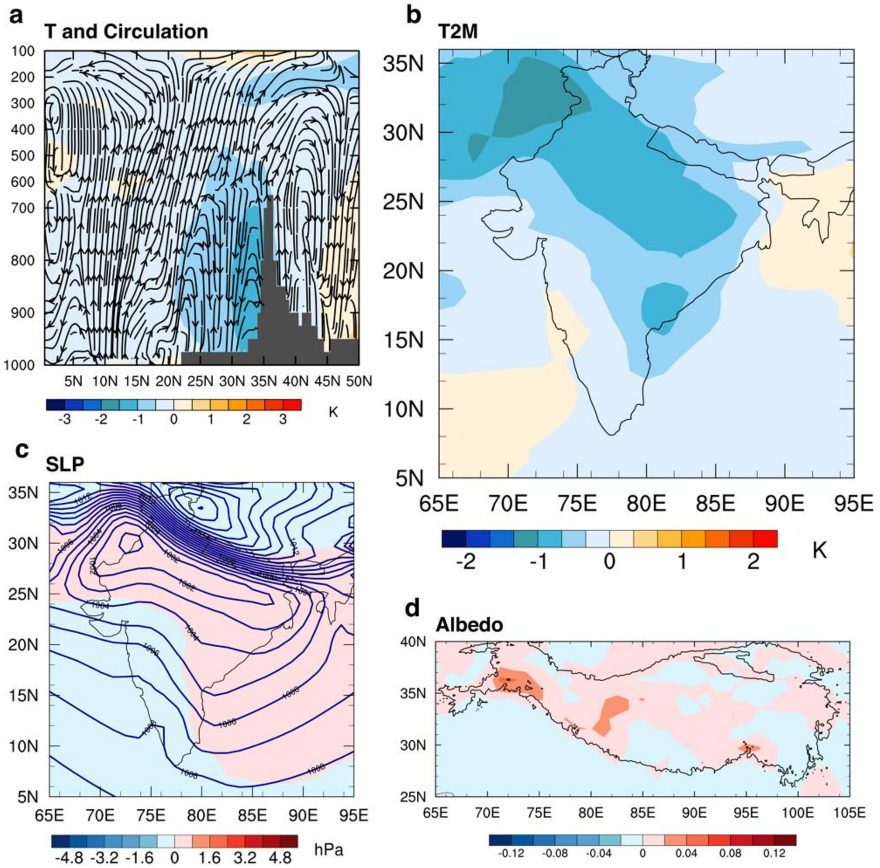


**Supplementary Figure 16. BC-reduction induced climate anomalies in April and May modeled by the COVID-19 case in comparison with the SSP case.** (**a**) Temperature and vertical circulation anomalies. (**b**) 2-meter temperature anomaly. (**c**) Sea level pressure (SLP) anomaly with superimposed SLP modeled by the COVID-19 experiment. (d) Surface albedo anomaly.

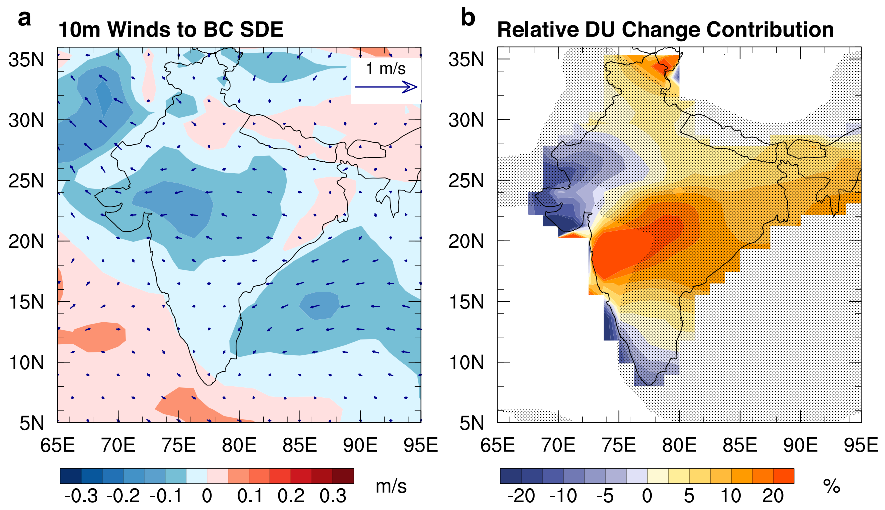


**Supplementary Figure 17. The contribution of snow-darkening effect to DU reduction.** (a) Difference in wind (vector) and wind speed (contour) between COVID-19 and NOSDE cases. (b) Relative contribution of SDE of BC to dust burden reduction, which is calculated as dust burden change of [COVID-19 - NOSDE] / [COVID-19 - SSP].

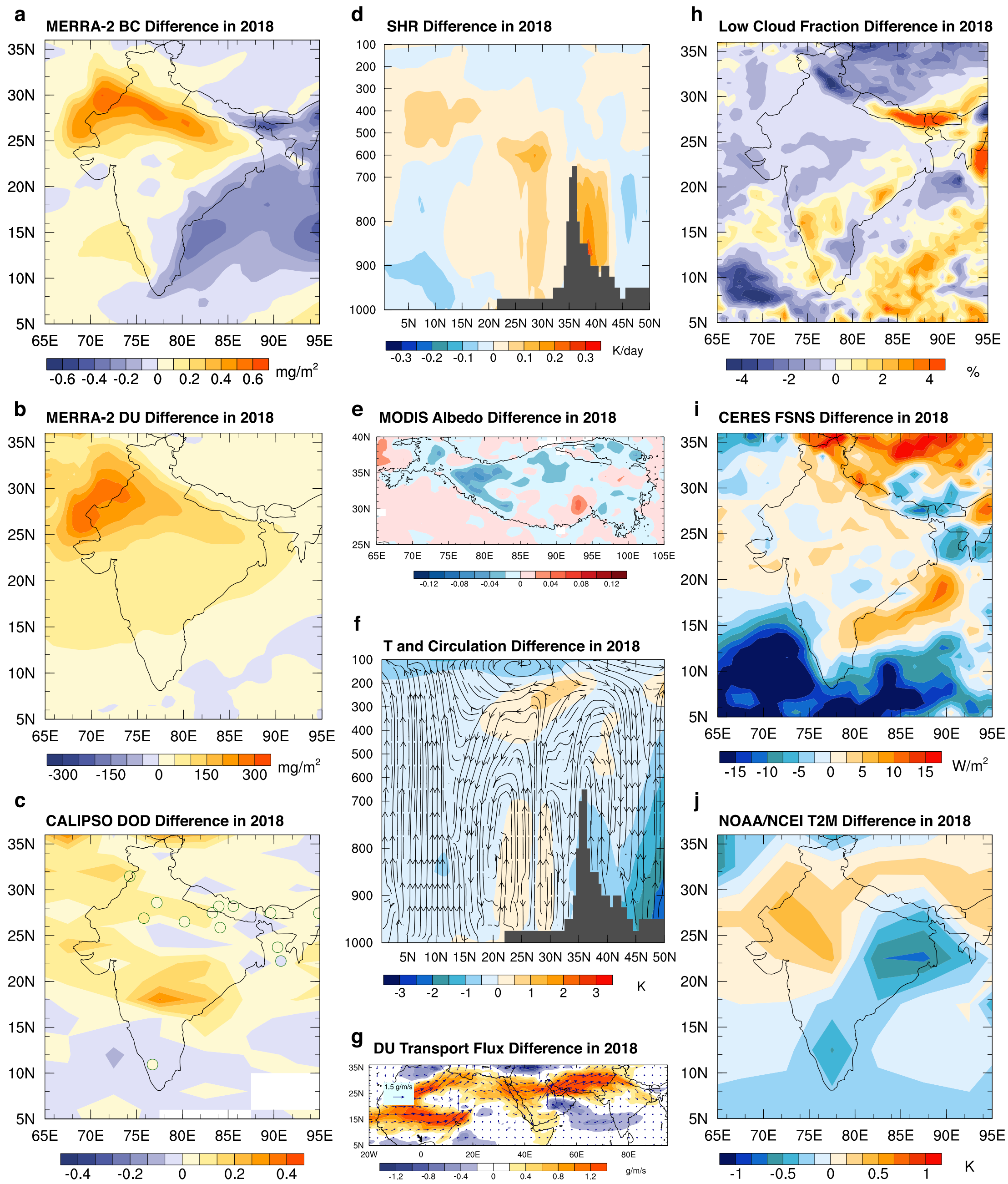


**Supplementary Figure 18. BC-induced anomalies in the premonsoon season of 2018 with high northern Indian DU loading.** (a) BC burdens in MERRA-2. (b) DU burdens in MERRA-2. (c) AOD of dust in CALIPSO (contour fill) and of coarse mode in AERONET (colored circles). (d) Zonal mean (70°E–90°E) shortwave heating rate in MERRA-2. (e) Surface albedo in MODIS. (f) Zonal mean (70°E–90°E) air temperature (contour fill) and stream function (vectors) in MERRA-2. (g) DU transport flux by zonal wind in MERRA-2. (h) Low cloud fraction in MERRA-2. (i) Surface shortwave cloud radiative effect in CERES. (j) 2-m air temperature in NOAA/NCEI. All anomalies are relative to 2015-2019 multiyear means.

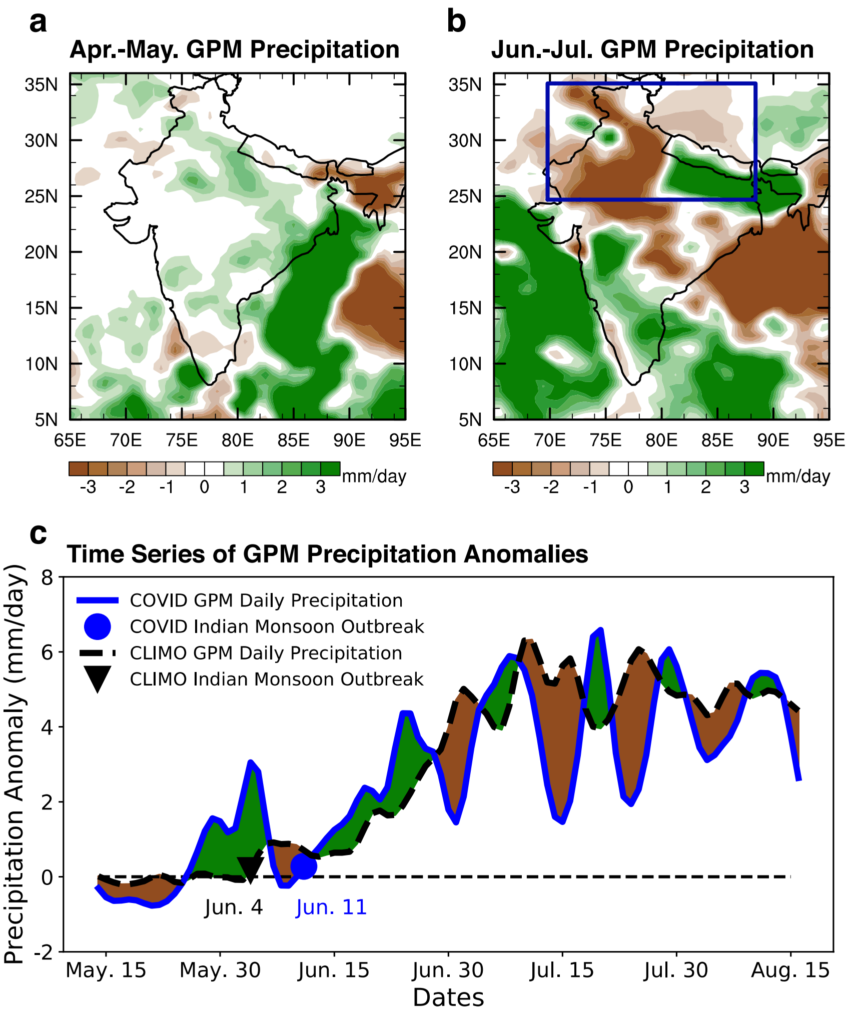


**Supplementary Figure 19. Differences of GPM precipitation for (a) April-May and (b) June-July between 2020 and 2015-2019 mean. (c) Time series of daily GPM precipitation anomalies (relative to the climatological mean of May) over northern India (70˚E~88˚E, 25˚E~35˚E as denoted by the square in b) in 2020 (blue) and 2015-2019 (black). Onsets of Indian monsoon outbreak are denoted by symbols.**

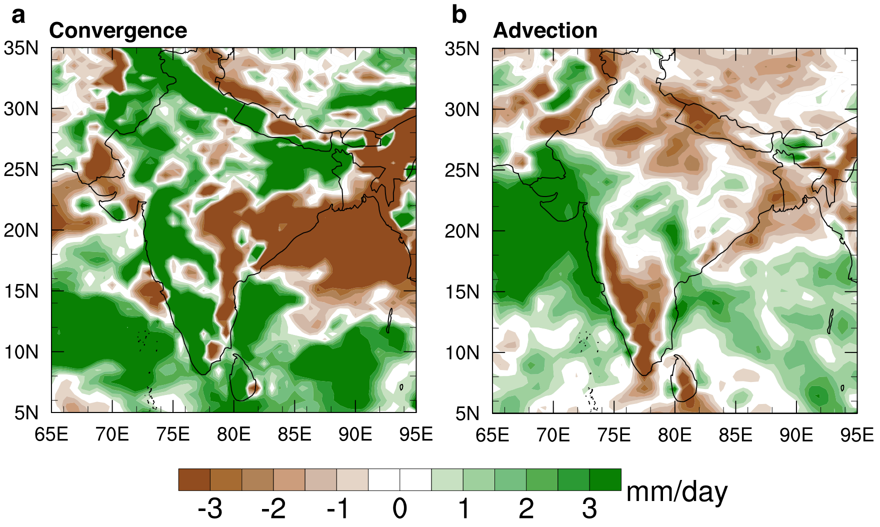


**Supplementary Figure 20. June-July differences of column integrated (a) moisture convergence and (b) moisture advection of MERRA-2 between 2020 and 2015-2019.**

**Supplementary References**


1. Granier, C. *et al.* The Copernicus Atmosphere Monitoring Service global and regional emissions (April 2019 version). Copernicus Atmosphere Monitoring Service https://dx.doi.org/10.24380/d0bn-kx16 (2019).
2. Doumbia, T. *et al.* Changes in global air pollutant emissions during the COVID-19 pandemic: a dataset for atmospheric modeling. *Earth Syst. Sci. Data* **13**, 4191-4206 (2021).
3. Wu, M. *et al.* Understanding processes that control dust spatial distributions with global climate models and satellite observations. *Atmos. Chem. Phys.* **20**, 13835-13855 (2020).
4. Luo, T. *et al.* Global dust distribution from improved thin dust layer detection using A-train satellite lidar observations. *Geophys. Res. Lett.* **42**, 620-628 (2015).
5. Luo, T. *et al.* Vertically resolved separation of dust and other aerosol types by a new lidar depolarization method. *Opt. Express* **23**, 14095-14107 (2015).
